# Cemented versus cementless cup fixation in total hip arthroplasty for proximal femoral fractures: analysis of revision and mortality rates from the German arthroplasty registry (EPRD)

**DOI:** 10.1186/s13018-025-06281-2

**Published:** 2025-10-08

**Authors:** Clemens Roitzsch, Cecilia Rogmark, Yinan Wu, Alexander Grimberg, Jörg Lützner, Anne Postler

**Affiliations:** 1 University Center of Orthopaedics and Traumatology, University Medicine Carl Gustav Carus Dresden, TU Dresden, Fetscherst. 74, 01307 Dresden, Germany; 2 Department of Orthopaedics, Skane University Hospital, Lund University, Malmö, Sweden; 3 German Arthroplasty Registry (EPRD), Berlin, Germany

**Keywords:** Hip fracture, Cemented cup, Cementless cup, Fracture total hip arthroplasty, German arthroplasty registry (EPRD)

## Abstract

**Background:**

The management of femoral neck fractures (FNF) in elderly patients depends on comorbidities, pre-fracture mobility, any hip joint disease, and life expectancy, with treatment typically involving either hemiarthroplasty (HA) or total hip arthroplasty (THA). While cemented femoral stem fixation is standard, there is no clear consensus regarding cemented versus cementless cup fixation in THA. This study aimed to compare revision and mortality rates between THA, divided into cemented and cementless cup fixation, and HA, following FNF.

**Methods:**

Data from the German Arthroplasty Registry (EPRD) were analyzed, including all patients with fracture-related THA or HA and available follow-up. A total of 34,501 patients undergoing THA (27,757 cementless, 6,744 cemented cups) and 72,022 patients with HA were included. 5-year revision and mortality rates were compared.

**Results:**

The 5-year revision rate was the lowest in the HA group (4.1%), followed by cemented cup THA (5.0%), and cementless cup THA (6.8%; *p* < 0.001). Dislocation, infection, and periprosthetic fracture were the leading causes of revision. The 5-year mortality rate was the lowest in cementless cup THA patients (23%), 43% in cemented cup THA patients and highest in HA patients (54%). Cementless fixation was associated with a higher revision risk (HR 1.28, 95% CI 1.14–1.44), while HA was associated with increased mortality (HR 1.26, 95% CI 1.22–1.31).

**Conclusion:**

Cemented cup fixation in THA after FNF is associated with lower revision rates but higher mortality compared to cementless fixation. In patients with limited life expectancy, HA remains the preferred option.

## Background

Hip fractures represent a major global health burden, particularly among the elderly, leading to high mortality and impaired quality of life and activities of daily living [1, 2]. Hip fractures are generally classified into two main categories: FNF and intertrochanteric fractures, each accounting for approximately 50%.

Intertrochanteric fractures are typically treated with closed reduction and intramedullary nailing [3, 4]. Displaced FNFs are managed with either joint replacement, generally favored as HA and THA in older adults with significantly decreased risk of reoperation and a better long-term hip function [5–7] or internal fixation, especially for younger patients. The decision to perform a THA or HA usually depends on patient-specific factors, including age and life expectancy, comorbidities, preoperative mobility and any preexisting symptomatic hip osteoarthritis (OA). Cementation of the femoral stem is very well investigated for elective hip arthroplasty. Based on international registries, multiple studies recommend cemented stem fixation for patients above 65–80 years, depending on the defined age cutoff, in elective THA as it reduces the risk of periprosthetic fracture and revision, while having no significant impact on mortality [8–11]. In patients with FNF one can expect poor bone quality regardless of age [12] and consequently, cemented femoral stem fixation is widely accepted as the preferred approach in THA and HA after FNFs, leading to lower risk of reoperation, lower pain and better mobility, but no increased mortality beyond the first postoperative days [13–16]. Whether non-cemented cups will carry an increased risk of periprosthetic acetabular or pelvic fracture is not known due to limited available evidence.

The optimal method of cup fixation in fracture THA remains controversial. Therefore, this study aimed to compare THA with cemented and cementless cup fixation, as well as HA, for the treatment for FNFs and to analyze their association on revision and mortality rates using data from the German Arthroplasty Registry (EPRD).

## Methods

### Study design

Data for this prospective observational cohort study were collected from the EPRD between November 1, 2012, and March 30, 2024. The STROBE guidelines were followed [17].

### Setting

The EPRD covers primary and revision arthroplasty surgeries for hip and knee. In collaboration of the German Society for Orthopaedics and Orthopaedic Surgery (DGOOC), the German Medical Technology Association (BVMed), the statutory health insurance funds (AOK Bundesverband GbR, Verband der Ersatzkassen e.V vdek), and more than 700 participating hospitals, over 2.5 million and approximately 70% of all hip and knee arthroplasties performed in Germany are gathered in this registry [18]. Data on revisions are recorded not only by hospitals but also by health insurance companies, ensuring near-complete follow-up. Besides elective procedures, emergency surgeries for the treatment of FNFs are also recorded in the EPRD.

### Patients

In this study, all patients registered in the EPRD under follow-up after receiving THA or HA for FNFs were included. Exclusion criteria were THA for other indication, such as OA, tumor or acetabular fracture.

### Variables and outcome measures

The registry provided patient characteristics such as age, sex, Body-Mass-Index (BMI) and Elixhauser Score [19]. The Elixhauser Score is a comorbidity index, calculated by patient’s diagnosis using the German version of the 10th International Classification of Diseases (ICD-10). Primary endpoints of the 3 groups (THA with cemented cup fixation, THA with cementless cup fixation, HA) were revision surgery and death. Reasons for early revision after 3 months, as well as revision and mortality rates after 5 and 10 years were analyzed. Revision reasons included infection, loosening and osteolysis (cup and or stem), periprosthetic fracture, dislocation, malalignment or others. The registry does not specify which implant components were revised or whether periprosthetic fractures occurred in the femur or acetabulum.

### Study size

Depending on patient’s age and comorbidities, either a HA or a THA is performed.

A total of 110,523 patients were included: 72,022 received HA, while 34,501 underwent THA (27,757 with a cementless cup and 6,744 with a cemented cup).

### Statistics

Statistical analysis was performed using R statistical software (Version R-4.2.0., Vienna, Austria). Descriptive statistics were calculated for the cemented and cementless cup fixation in THA as well as HA group. Continuous variables were reported as medians with interquartile ranges, while categorical variables were summarized as frequencies and percentages. Group comparisons for continuous data were conducted using the Kruskal-Wallis rank sum test, and categorical data were analyzed using the Chi-square test. Cumulative incidences rate with 95% log-log confidence interval for the endpoints of arthroplasty revision and patient mortality with 8-year follow-up were determined using the Kaplan–Meier estimator. To examine the impact of various patient characteristics and type of surgery on revision and mortality outcomes, Hazard Ratios (HRs) were calculated using multivariate Cox regressions analyses. The covariates included age at the time of the first surgery, sex, BMI, the Elixhauser Comorbidity Score at the time of the first surgery, annual numbers of elective hip surgery by hospital. A *p*-value threshold of 0.05 was considered indicative of statistical significance.

## Results

Female patients predominated in all 3 groups. Median age in the HA group was 85 years, 81 for cemented and 74 for the cementless cup group (Table 1).

**Table 1:** Patient characteristics

Characteristic	Cemented N = 6744^1^	Cementless N = 27,757^1^	Hemi N = 72,022^1^
Age	81 (75, 85)	74 (67, 80)	85 (80, 89)
*Sex*			
Female	4995 (74%)	18,853 (68%)	51,434 (71%)
Male	1749 (26%)	8904 (32%)	20,588 (29%)
*BMI*			
Underweight[< 18.5 kg/m^2^]	261 (3.9%)	923 (3.3%)	3052 (4.2%)
Normal[18.5–24.99 kg/m^2^]	2648 (39%)	11,220 (40%)	30,878 (43%)
Pre-obese[25.0–29.99 kg/m^2^]	1662 (25%)	7639 (28%)	18,205 (25%)
Obese 1[30.0–34.99 kg/m^2^]	398 (5.9%)	2097 (7.6%)	4474 (6.2%)
Obese 2[35.0–39.99 kg/m^2^]	72 (1.1%)	441 (1.6%)	786 (1.1%)
Obese 3[> = 40 kg/m^2^]	31 (0.5%)	136 (0.5%)	214 (0.3%)
Missing	1672 (25%)	5301 (19%)	14,413 (20%)
Elixhauser score	3 (1, 4)	2(1, 3)	3(2, 4)

^1^Median (Q1, Q3); n (%)

### Revision rates

At the 5-year follow-up, HA had the lowest revision rate (2,988/72,022, 4.1%), whereas THA with a cementless cup exhibited the highest revision rate (1,887/27,757, 6.8%). THA with cemented cup fixation showed an intermediate revision rate of 5% (336/6,744). Most common were dislocation, infection and periprosthetic fracture, with comparable rates for dislocation and infection in all 3 groups, while fracture rates were twice as high in the cementless compared to the cemented and HA group (14% vs 7% and 7.9%) (Table 2). In over 30% of cases, the reason for revision was unknown. No cases of isolated cup revision were recorded.

**Table 2:** All-time reasons for revision

Characteristic	Cemented N = 344^1^	Cementless N = 1909^1^	Hemi N = 3005^1^
*Reason*			
Component failure	1 (0.3%)	7 (0.4%)	9 (0.3%)
Dislocation	72 (21%)	350 (18%)	555 (18%)
Infection	68 (20%)	326 (17%)	796 (27%)
Loosening (Cup and stem)	3 (0.9%)	10 (0.5%)	6 (0.2%)
Loosening (Cup)	14 (4.1%)	98 (5.1%)	13 (0.4%)
Loosening (Stem)	13 (3.8%)	86 (4.5%)	48 (1.6%)
Malalignment	2 (0.6%)	40 (2.1%)	23 (0.8%)
Missing	130 (38%)	603 (32%)	1088 (36%)
Other reasons	17 (5%)	110 (5.7%)	220 (7.4%)
Periprosthetic fracture	24 (7.0%)	276 (14%)	236 (7.9%)
Progression of arthrosis	0 (0%)	0 (0%)	7 (0.2%)
Wear	0 (0%)	3 (0.2%)	4 (0.1%)

^1^n (%)

**Fig. 1:**
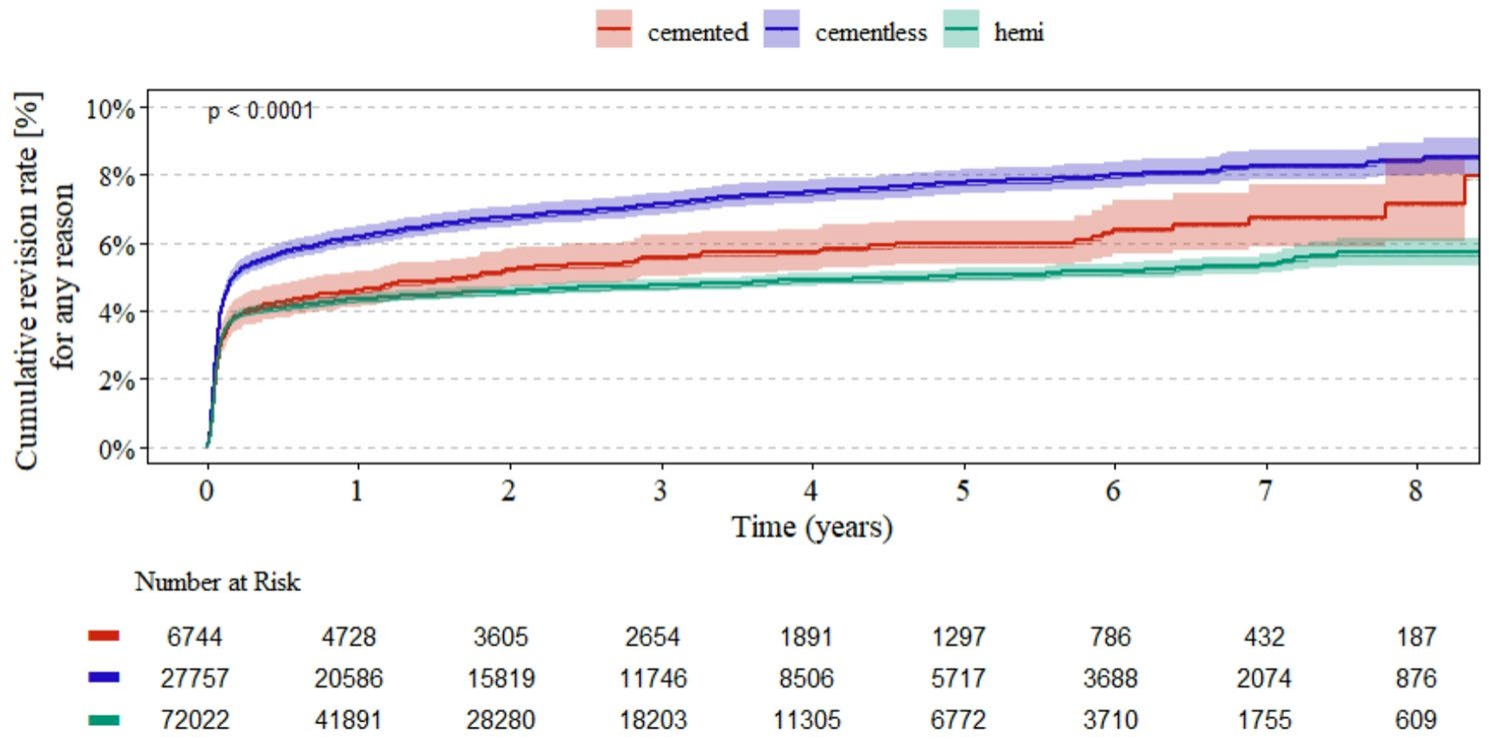
Kaplan–Meier survival analysis for revision rate over time for HA (hemi), cemented and cementless cup fixation

Cementless cup fixation had consistently the highest revision rates (Fig. 1). Cementless cups were associated with an increased risk of revision compared to cemented cups (HR 1.28, 95% CI 1.14–1.44, *p* < 0.001) (Table 3). Higher revision rates were also found for male gender (HR 1.17, 95% CI 1.1–1.24, *p* < 0.001), higher Elixhauser Score (HR 1.15, 95% CI 1.13–1.16, *p* < 0.001) and higher BMI, with the risk increasing exponentially with higher BMI (BMI 30–35 kg/m^2^ HR 1.24, 95% CI 1.11–1.37 vs BMI > 40 kg/m^2^ HR 2.43, 95% CI 1.86–3.18, *p* < 0.001).

**Table 3:** Cox regression analysis for revision risk

Characteristic	HR^1^	95% CI^1^	*p*-value
*Group*			
THA cemented cup (reference)	–	–	
THA cementless cup	1.28	1.14, 1.44	< 0.001
HA	0.89	0.80, 1.00	0.052
*Sex*			
Female (reference)	–	–	
Male	1.17	1.10, 1.24	< 0.001
Age per year	0.99	0.98, 0.99	< 0.001
Elixhauser Score per 1	1.15	1.13, 1.16	< 0.001
*BMI*			
Normal[18.5–24.99 kg/m^2^] (reference)	–	–	
Underweight[< 18.5 kg/m^2^]	0.95	0.82, 1.12	0.6
Pre-obese[25.0–29.99 kg/m^2^]	1.04	0.97, 1.12	0.2
Obese 1[30.0–34.99 kg/m^2^]	1.24	1.11, 1.37	< 0.001
Obese 2[35.0–39.99 kg/m^2^]	1.66	1.38, 2.00	< 0.001
Obese 3[> = 40 kg/m^2^]	2.43	1.86, 3.18	< 0.001

^1^HR = Hazard ratio; CI = Confidence interval

### Mortality rates

**Fig. 2:**
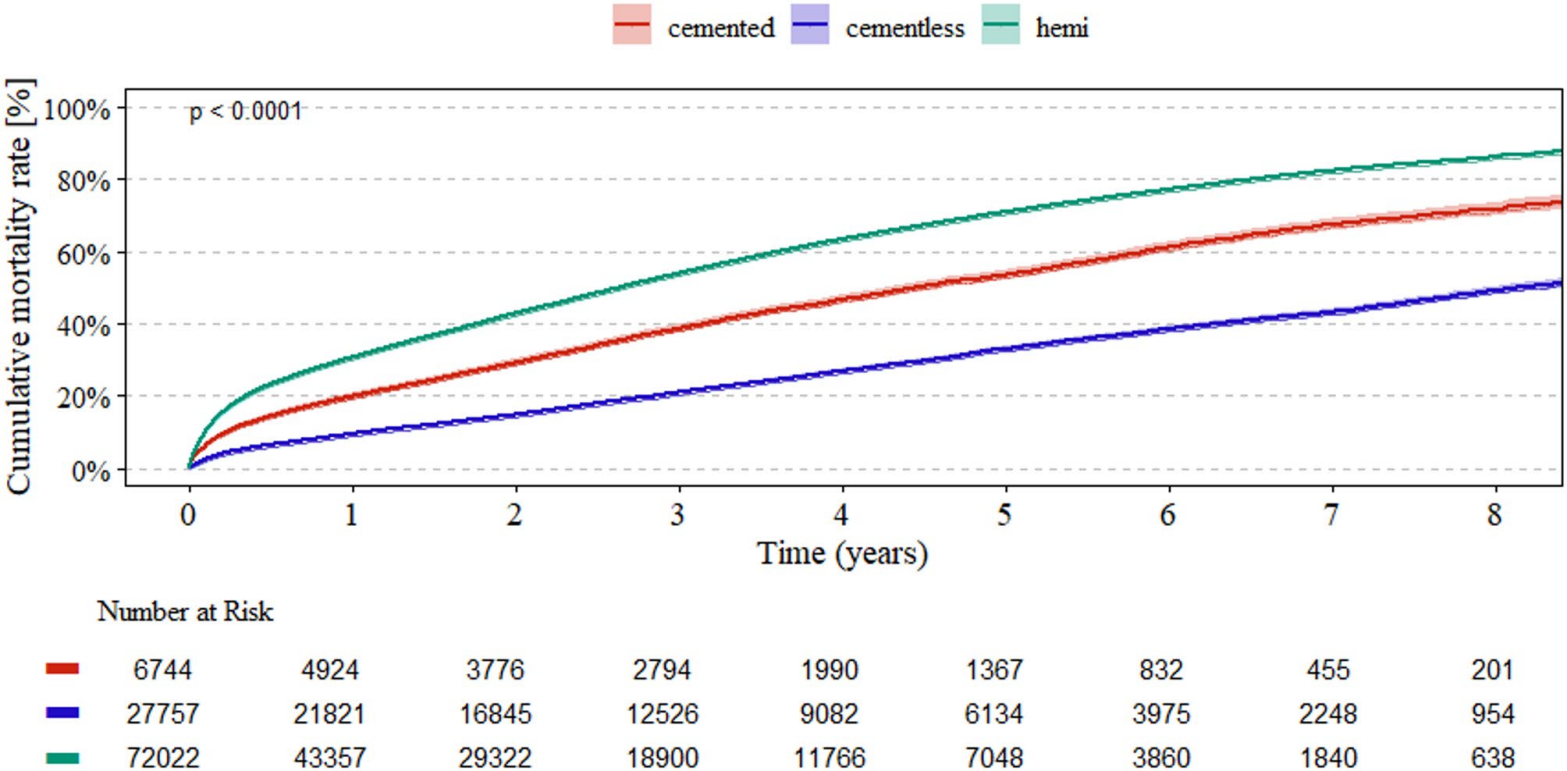
Kaplan–Meier survival analysis for mortality rates over time for HA (hemi), cemented and cementless cup fixation

Mortality rates after 5 years were highest in the HA group (39,085/72,022, 54%), followed by cemented cup fixation THA (2,878/6,744, 43%), while the lowest rate was observed in cementless cup THA (6,294/27,757, 23%,) (*p* < 0.001). The survival analysis showed consistently lower mortality in the cementless THA group (Fig. 2). Cox regression analysis showed higher mortality for HA (HR 1.26, 95% CI 1.22–1.31, *p* < 0.001), male gender (HR 1.73, 95% CI 1.7–1.77, *p* < 0.001), higher age (HR 1.05, 95% CI 1.05–1.05, *p* < 0.001), higher Elixhauser Score (HR 1.15, 95% CI 1.14–1.15, *p* < 0.001), and underweight (HR 1.53, 95% CI 1.46–1.6, *p* < 0.001) (Table 4). Overweight and mild obesity up to a BMI < 40 kg/m^2^ had a positive effect on survival. Cementless cup fixation was associated with lower mortality (HR 0.67, 95% CI 0.64–0.7, *p* < 0,001).

**Table 4:** Cox regression analysis for mortality

Characteristic	HR^1^	95% CI^1^	*p*-value
*Group*			
THA cemented cup (reference)	–	–	
THA cementless cup	0.67	0.64, 0.70	< 0.001
HA	1.26	1.22, 1.31	< 0.001
*Sex*			
Female (reference)	–	—	
Male	1.73	1.70, 1.77	< 0.001
Age per year	1.05	1.05, 1.05	< 0.001
Elixhauser Score per 1	1.15	1.14, 1.15	< 0.001
*BMI*			
Normal[18.5–24.99 kg/m^2^] (reference)	–	–	
Underweight[< 18.5 kg/m^2^]	1.53	1.46, 1.60	< 0.001
Pre-obese[25.0–29.99 kg/m^2^]	0.82	0.80, 0.84	< 0.001
Obese 1[30.0–34.99 kg/m^2^]	0.73	0.70, 0.76	< 0.001
Obese 2[35.0–39.99 kg/m^2^]	0.78	0.72, 0.86	< 0.001
Obese 3[> = 40 kg/m^2^]	1.01	0.86, 1.18	> 0.9

^1^HR = Hazard ratio; CI = Confidence interval

## Discussion

This study analyzed 5-year revision and mortality rate following cemented or cementless cup fixation in THA and HA for FNFs, using data from the EPRD. Cemented cup fixation was associated with lower revision rates compared to cementless cups but was also linked to higher mortality. In contrast, HA exhibited the lowest revision rates while having the highest mortality rates.

To the best of the author’s knowledge, this is the first large-scale study to compare long-term revision and mortality rates for cemented and cementless cup fixation in THA following FNFs.

The lower revision and increased mortality rates observed in HA patients are most likely attributable to a selection bias, where older individuals with higher burden of comorbidities are chosen for HA treatment. This can both result in reduced mobility and thereby lesser risk of acetabulum erosion and a reluctancy to perform revision surgery when a complication develops. HA is a less complex surgical procedure than THA with shorter operation time, reduced blood loss and lower dislocation rates [20]. A meta-analysis comparing THA and HA following FNF reported a better function and quality of life after THA, along with a reduced risk for revision, while overall mortality remained comparable. However, the subanalysis in this study of patients over 80 years showed no difference in 1-year mortality, hip function, pain and reoperation rate between the 2 groups [21]. In this context, increased mortality rates in HA patients in this study may be partly explained by their high median age of 85 years, the 5- year follow up period and the fact that HA is often chosen for frail patients with significant comorbidities.

Regarding cup fixation, most research focuses on primary THA for OA. Due to a lower risk of revision for any reason regardless of age, cemented cup fixation, described as more reliable beyond the first postoperative decade [22] and considered as gold standard [23], is preferred.

An analysis of national hip arthroplasty registries from 7 countries with revision as endpoint showed a reduced revision risk for cemented (cup and stem) versus cementless THA across all countries involved in the study. Additionally, fully cemented fixation outperformed hybrid fixation (cementless cup and cemented stem) in all but one country [10].

These findings align with our study, demonstrating lower revision rates for cemented cup fixation compared to cementless cup fixation, although our cohort exclusively included fracture THA. The available literature regarding specific causes of revision following cup cementation in FNF remains limited. We found dislocation, infection, and periprosthetic fracture to be the most common reasons for revision. Notably, cementless cup fixation was associated with a 2-fold higher rate of fractures compared to cemented cups and HA. Consistent with our findings, the studies included in a 2011 meta-analysis did not reveal any significant differences in rates of dislocation or infection between cemented and cementless cup fixation [24]. However, it is important to acknowledge that most available studies either did not report specific revision causes or defined aseptic loosening as the primary endpoint. Furthermore, these studies investigated cemented cup fixation in the setting of primary THA for OA, not fracture-related THA. In contrast, patients in our cohort undergoing THA for FNF were older, more comorbid as indicated by a higher Elixhauser Score, and likely had inferior bone quality compared to patients undergoing primary THA for OA. Consequently, FNF patients are at increased risk for periprosthetic fractures. In contrast to cup cementation, stem fixation in FNF is more thoroughly investigated. Cemented stems have been shown to reliably reduce the risk of periprosthetic fractures and are recommended by international guidelines in the context of fracture THA [13, 14]. A recently published multicenter randomized controlled trial comparing cemented and cementless stems in HA for FNF demonstrated significantly improved quality of life and a reduced risk for periprosthetic fracture for cemented stem fixation after 12 months follow up in uncemented versus cemented HA for periprosthetic fracture [25]. Apart from periprosthetic fractures, contralateral refractures also play an important role in geriatric patients. Risk factors include osteoporosis, advanced age, and female sex [26].

Besides, none of the mentioned studies investigated mortality differences between cemented and cementless cup fixation. In a large matched cohort study of 170.000 patients with cemented or cementless primary THA due to OA and 860.000 population-based controls, a subanalysis of hybrid THA indicated that cemented femoral components were associated with an increased early mortality, while cemented cups with a cementless femur were not linked to higher mortality. The authors concluded that cementing the femoral component is more dangerous than acetabular, most likely because of the higher risk of bone cement implantation syndrome (BCIS) [27]. A prospective multicenter study from Denmark showed significantly less inhouse complications and pulmonary embolisms after cementless versus hybrid and cemented primary THA in elderly patients but no difference in mortality after 30 days [8]. BCIS is a well-recognized complication of cemented THA, potentially leading to embolisms and increased early mortality but more frequently associated with cemented femoral fixation than with cemented acetabular components [28]. In this study, cementless cup fixation was associated with a significantly lower mortality compared to cemented fixation technique. Given that the cemented group was, on average, 8 years older and had a higher Elixhauser Score, this finding may be influenced by selection bias. We fail to see how cementless instead of cemented cup fixation should be the solely reason for the observed reduction in early as well as long-term mortality. However, mortality rates in FNF patients are reported to be high with 18% after 90 days, so comparison between elective arthroplasty and arthroplasty following FNF regarding mortality is complicated [29]. Importantly, several studies have demonstrated that geriatric co-management can positively influence outcomes in patients with hip fractures and reduce mortality and adverse effects [30–32].

Some limitations need to be noted. In general, registry-based studies lack detailed information about the patients included. That is why the concrete decision-making process for the revision and the operative treatment were unknown. Patient´s comorbidities and life expectancy are the main factors for decision making between HA, cemented and cementless THA, so this constitutes a source for potential selection bias. Moreover, only comorbidities and risk factors documented in the EPRD were included in the adjustment and cox regression analysis. However, these do not necessarily reflect all existing and clinically relevant comorbid conditions. Furthermore, the reason for revision was not documented in over 30% of cases, which may affect the accuracy of our findings. Additionally, only revision and mortality rates were analyzed, neither addressing confounders such as type of implants nor peri- and postoperative complications that did not lead to revision. Furthermore, outcome parameters like function, satisfaction or ambulation are not entered into the registry, but play a role in the clinical shared-decision making. Another important limitation is that only the cup fixation method was assessed, but it is unknown if the femoral component was cementless or cemented. Finally, while the German Arthroplasty Registry (EPRD) covers approximately 70% of all arthroplasties performed in Germany, it remains a voluntary registry, meaning not all THA and HA procedures were included. Nonetheless, we consider the large sample size to ensure that our findings are broadly representative.

## Conclusion

For patients with hip fractures, cemented cup fixation in THA should be considered due to its lower revision rates compared to uncemented fixation. Additional studies are needed to refine recommendations for cup fixation in fracture-related THA, considering both revision risk and overall survival outcomes. The difference in mortality between treatment options may to a certain extent be explained by selection bias.

HA was associated with the lowest revision rate and remains an appropriate choice for frail elderly patients.

## Data Availability

The datasets used and/or analysed during the current study are available from the corresponding author on reasonable request.

## Abbreviations

THA: Total hip arthroplasty
HA: Hemiarthroplasty
FNF: Femoral neck fracture
OA: Osteoarthritis
EPRD: German arthroplasty registry (Endoprothesenregister Deutschland)
BMI: Body mass index
ICD-10: International classification of diseases, 10th Revision
HR: Hazard ratio
CI: Confidence interval
BCIS: Bone cement implantation syndrome

## References

[1] Chen YP, Kuo YJ, Hung SW, Wen TW, Chien PC, Chiang MH. Loss of skeletal muscle mass can be predicted by sarcopenia and reflects poor functional recovery at one year after surgery for geriatric hip fractures. Injury. 2021;52(11):3446–52. 10.1016/j.injury.2021.08.007 34404509

[2] Maffulli N, Aicale R. Proximal femoral fractures in the elderly: a few things to know, and some to forget. Medicina (Kaunas). 2022;58(10):1314. 10.3390/medicina58101314 PMC9612001 36295475

[3] Marsillo E, Pintore A, Asparago G, Oliva F, Maffulli N. Cephalomedullary nailing for reverse oblique intertrochanteric fractures 31A3 (AO/OTA). Orthop Rev (Pavia). 2022;14(6):38560. 36267220 10.52965/001c.38560 PMC9568432

[4] Gargano G, Poeta N, Oliva F, Migliorini F, Maffulli N. Zimmer natural nail and ELOS nails in pertrochanteric fractures. J Orthop Surg Res. 2021;16(1):509. 10.1186/s13018-021-02634-9 PMC8371819 34407829

[5] Deng J, Wang G, Li J, Wang S, Li M, Yin X. A systematic review and meta-analysis comparing arthroplasty and internal fixation in the treatment of elderly displaced femoral neck fractures. OTA Int. 2021;4(1):e087. 10.1097/OI9.0000000000000087 PMC8016607 33937715

[6] Parker MJ. Hemiarthroplasty versus internal fixation for displaced intracapsular fractures of the hip in elderly men: a pilot randomised trial. Bone Joint J. 2015;97–B(7):992–6. 26130358 10.1302/0301-620X.97B7.35524

[7] Heetveld MJ, Rogmark C, Frihagen F, Keating J. Internal fixation versus arthroplasty for displaced femoral neck fractures: What is the evidence? J Orthop Trauma. 2009;23(6):395–402. 10.1097/BOT.0b013e318176147d 19550224

[8] Lindberg-Larsen M, Petersen PB, Jorgensen CC, Overgaard S, Kehlet H, Madsen F. Postoperative 30-day complications after cemented/hybrid versus cementless total hip arthroplasty in osteoarthritis patients >70 years A multicenter study from the Lundbeck Foundation Centre for Fast-track Hip and Knee replacement database and the Danish Hip Arthroplasty Register. Acta Orthop. 2020;91(3):286–92. 10.1080/17453674.2020.1745420 PMC8023910 32285735

[9] Morlock M, Perka C, Melsheimer O, Kirschbaum SM. Influence of the type of stem and its fixation on revision and immediate postoperative mortality in elective total hip arthroplasty. Bone Joint J. 2024;106-B(3 Supple A):130–6. 10.1302/0301-620X.106B3.BJJ-2023-0820.R2 38423088

[10] Troelsen A, Malchau E, Sillesen N, Malchau H. A review of current fixation use and registry outcomes in total hip arthroplasty: the uncemented paradox. Clin Orthop Relat Res. 2013;471(7):2052–9. 10.1007/s11999-013-2941-7 PMC3676623 23539124

[11] Gonzalez Della Valle A, Odum SM, De A, Barrington JW, Huddleston JI, Illgen RL. The effect of femoral fixation on revision and mortality following elective total hip arthroplasty in patients over the age of 65 years. An analysis of the American joint replacement registry. J Arthroplasty. 2022;37(6):1105–10. 10.1016/j.arth.2022.01.088 35131391

[12] Strøm Rönnquist S, Viberg B, Kristensen MT, Palm H, Jensen JEB, Madsen CF. Frailty and osteoporosis in patients with hip fractures under the age of 60-a prospective cohort of 218 individuals. Osteoporos Int. 2022;33(5):1037–55. 10.1007/s00198-021-06281-y PMC9007814 35029719

[13] Overview | Hip fracture. management | Guidance | NICE [Internet]. NICE; 2011 [zitiert 20. März 2025]. Verfügbar unter: https://www.nice.org.uk/guidance/cg124

[14] O’Connor MI, Switzer JA, AAOS Clinical Practice Guideline Summary. Management of hip fractures in older adults. J Am Acad Orthop Surg. 2022;30(20):e1291–6. 36200817 10.5435/JAAOS-D-22-00125

[15] Parker MJ, Chatterjee R, Onsa M, Cawley S, Gurusamy K. Cemented versus uncemented hemiarthroplasty for displaced intracapsular fractures of the hip. Bone Joint J. 2023;105–B(11):1196–200. 37907087 10.1302/0301-620X.105B11.BJJ-2023-0534.R1

[16] Lewis SR, Macey R, Parker MJ, Cook JA, Griffin XL. Arthroplasties for hip fracture in adults. Cochrane Database of Systematic Reviews [Internet]. 2022 [zitiert 10. April 2025];(2). Verfügbar unter: https://www.cochranelibrary.com/cdsr/doi/10.1002/14651858.CD013410.pub2/full?utm_source=chatgpt.com 10.1002/14651858.CD013410.pub2 PMC8841979 35156194

[17] von Elm E, Altman DG, Egger M, Pocock SJ, Gøtzsche PC, Vandenbroucke JP. The strengthening the reporting of observational studies in epidemiology (STROBE) statement: guidelines for reporting observational studies. Lancet. 2007;370(9596):1453–7. 10.1016/S0140-6736(07)61602-X 18064739

[18] https://www.eprd.de/fileadmin/user_upload/Dateien/Publikationen/Berichte/AnnualReport2024-Web_2025-03-27_F.pdf [Internet]. EPRD Annual Report 2024.

[19] van Walraven C, Austin PC, Jennings A, Quan H, Forster AJ. A modification of the elixhauser comorbidity measures into a point system for hospital death using administrative data. Med Care. 2009;47(6):626. 19433995 10.1097/MLR.0b013e31819432e5

[20] Keating JF, Grant A, Masson M, Scott NW, Forbes JF. Displaced intracapsular hip fractures in fit, older people: a randomised comparison of reduction and fixation, bipolar hemiarthroplasty and total hip arthroplasty. Health Technol Assess. 2005;9(41):iii–iv, ix–x, 1–65. 10.3310/hta9410 16202351

[21] Lewis DP, Wæver D, Thorninger R, Donnelly WJ. Hemiarthroplasty vs total hip arthroplasty for the management of displaced neck of femur fractures: a systematic review and meta-analysis. J Arthroplasty. 2019;34(8):1837–e18432. 31060915 10.1016/j.arth.2019.03.070

[22] Toossi N, Adeli B, Timperley AJ, Haddad FS, Maltenfort M, Parvizi J. Acetabular components in total hip arthroplasty: Is there evidence that cementless fixation is better? J Bone Joint Surg Am. 2013;95(2):168–74. 23324965 10.2106/JBJS.K.01652

[23] Van Praet F, Mulier M. To cement or not to cement acetabular cups in total hip arthroplasty: a systematic review and re-evaluation. SICOT J. 2019;5:35. 31571579 10.1051/sicotj/2019032 PMC6771226

[24] Pakvis D, van Hellemondt G, de Visser E, Jacobs W, Spruit M. Is there evidence for a superior method of socket fixation in hip arthroplasty? A systematic review. Int Orthop. 2011;35(8):1109–18. 21404024 10.1007/s00264-011-1234-6 PMC3167434

[25] Fernandez MA, Achten J, Parsons N, Griffin XL, Png ME, Gould J. Cemented or uncemented hemiarthroplasty for intracapsular hip fracture. New Engl J Med. 2022;386(6):521–30. 10.1056/NEJMoa2108337 35139272

[26] Chen M, Li Y, Yang Y, Zhuang W. Analysis of the risk factors for contralateral refracture after hip fracture surgery in elderly individuals: a retrospective study. J Orthop Surg Res. 2024;19(1):681. 39438923 10.1186/s13018-024-05177-x PMC11515634

[27] Garland A, Gordon M, Garellick G, Kärrholm J, Sköldenberg O, Hailer NP. Risk of early mortality after cemented compared with cementless total hip arthroplasty: a nationwide matched cohort study. Bone Joint J. 2017;99–B(1):37–43. 10.1302/0301-620X.99B1.BJJ-2016-0304.R1 28053255

[28] Donaldson AJ, Thomson HE, Harper NJ, Kenny NW. Bone cement implantation syndrome. Br J Anaesth. 2009;102(1):12–22. 10.1093/bja/aen328 19059919

[29] Postler A, Posten C, Schubert M, Beyer F, Lützner J, Vicent O. Patients risk for mortality at 90 days after proximal femur fracture–A retrospective study in a tertiary care hospital. BMC Geriatr. 2024;24(1):130. 10.1186/s12877-024-04733-8 PMC10838409 38310209

[30] Neuerburg C, Förch S, Gleich J, Böcker W, Gosch M, Kammerlander C. [internet].proved outcome in hip fracture patients in the aging population following co-managed care compared to conventional surgical treatment: a retrospective, dual-center cohort study. BMC geriatrics [Internet]. 27. November 2019 [zitiert 20. Juni 2023];19(1). Verfügbar unter: https://pubmed.ncbi.nlm.nih.gov/31775659/?dopt=Abstract 10.1186/s12877-019-1289-6 PMC6880371 31775659

[31] Liu T, Zhang X, Zhang J, Ye P, Yang M, Tian M. Effect of the orthogeriatric co-management on older hip fracture patients with multimorbidity: a post-hoc exploratory subgroup analysis of a non-randomised controlled trial. J Orthop Surg Res. 2024;19(1):780. 10.1186/s13018-024-05263-0 PMC11580192 39574198

[32] Quaranta M, Miranda L, Oliva F, Migliorini F, Pezzuti G, Maffulli N. Haemoglobin and transfusions in elderly patients with hip fractures: the effect of a dedicated orthogeriatrician. J Orthop Surg Res. 2021;16:387. 34134743 10.1186/s13018-021-02524-0 PMC8207795

